# Predicting Brazilian Court Decisions

**DOI:** 10.7717/peerj-cs.904

**Published:** 2022-03-25

**Authors:** André Lage-Freitas, Héctor Allende-Cid, Orivaldo Santana, Lívia Oliveira-Lage

**Affiliations:** 1 Universidade Federal de Alagoas, Maceió, Brazil; 2 JusPredict, Salvador, Brazil; 3 Pontificia Universidad Católica de Valparaíso, Valparaíso, Chile; 4 Universidade Federal do Rio Grande do Norte, Natal, Brazil; 5 Procuradoria Geral do Estado de Alagoas, Maceió, Brazil

**Keywords:** Legal informatics, Litigation prediction, Legal outcome forecast, Predictive algorithms, Jurimetrics, Law, Legal, Machine learning, Artificial intelligence

## Abstract

Predicting case outcomes is useful for legal professionals to understand case law, file a lawsuit, raise a defense, or lodge appeals, for instance. However, it is very hard to predict legal decisions since this requires extracting valuable information from myriads of cases and other documents. Moreover, legal system complexity along with a huge volume of litigation make this problem even harder. This paper introduces an approach to predicting Brazilian court decisions, including whether they will be unanimous. Our methodology uses various machine learning algorithms, including classifiers and state-of-the-art Deep Learning models. We developed a working prototype whose F1-score performance is ~80.2% by using 4,043 cases from a Brazilian court. To our knowledge, this is the first study to present methods for predicting Brazilian court decision outcomes.

## Introduction

Legal systems have been trying to improve legal certainty by publishing statutes and court opinions. In addition to publishing the laws, legal systems usually provide further support to legal certainty through judicial decisions. These decisions might be useful not only to adjudicate specific situations but also to influence social behavior by affirming the legal consequences of a person’s actions (Starr, 2014). Predicting legal decisions is thus fundamental to both understanding the consequences of behavior and to improving the quality of legal work product.

In Brazil for example, lower court decisions (*Sentenças*) might be appealed to Brazilian courts (*Tribiunais de Justiça*) to be reviewed by second instance court judges. In an appellate court, judges decide together upon a case, and their decisions are compiled in judgement reports (*Acórdãos*).

These reports are very useful for understanding jurisprudence generally, and provide guidance for lawyers and other court members about these decisions. For instance, attorneys often use these documents to prepare cases, while judges would do well to at least consider these reports, if not use them as guidelines, given the Civil Procedure Code enacted in 2015 do Brasil (2015) ensures by law that jurisprudence should expressly be taken into account by the courts when deciding a case. Scholars have been investigating how this compliance can be achieved, to avoid arbitrary dispensation of justice as discussed in Serra Júnior (2017), Silva & de Lima (2018), Tavares (2018), da Gama & Medeiros (2017).

A common, critical task for litigators is to identify likely rulings based on the specific court and the facts of the case, as discussed by Loevinger (1963), to optimize arguments to achieve the most favorable outcome. Although attorneys can find relevant information in public Acórdãos, the myriad available documents make this task very complex and error-prone, even for experienced lawyers. In order to extract information regarding the Acórdãos, one must read each, including the summary to ascertain the subject, then the decision report to determine the vote of each judge, and the final decision, noting whether it was unanimous. Moreover, each Acórdão might contain multiple cases and decisions, increasing its complexity. This problem is compounded by the fact that there usually are hundreds–and sometimes thousands–of Acórdãos related to the case at hand.

In addition to Brazil, several other legal systems in the world share the very same problem of predicting legal decisions. The challenge is hence generalized as *how to automatically predict legal decisions with a satisfactory level of accuracy* to support the work of attorneys, judges, and other professionals such as accountants and realtors. By “satisfactory”, we mean that the quality of the prediction in terms of accuracy should be comparable–or even higher–than one made by legal experts.

Though computers have been used for decades to address the challenge Loevinger (1963), predicting legal decision outcomes with requisite accuracy is not a trivial task. For instance, in Ashley & Brüninghaus (2009), propose a method for classifying and predicting cases that attains 91.8% accuracy; however, the evaluation relies on a small data set of only 146 cases. Katz, Bommarito & Blackman (2017) use historical data to predict US Supreme Court decisions by classifying decisions into three categories, and by presenting judge profiles. Their approach achieves 70.2% accuracy using a data set of 28,000 cases. Also using US Supreme Court data, Ruger et al. (2004) compare the predictive performance of legal experts to a trained statistical model, using fewer than 200 cases.

Aletras et al. (2016) use Support-Vector Machine (SVM) to predict whether cases from the European Court of Human Rights would be decided as violating Articles 3, 6, and 8 of the European Convention on Human Rights. Their results achieved 78% accuracy on 584 European Court cases separated by subjects. Chalkidis, Androutsopoulos & Aletras (2019) use language models based on artificial neural networks to predict whether any of the 66 Articles or Protocols of the European Convention of Human Rights was violated (∼82% F1 score). Moreover, the authors also predict which Articles or Protocols were violated (∼60% F1 score) and they classify the cases regarding their importance by using a regression algorithm. The data set used in Chalkidis, Androutsopoulos & Aletras (2019) has ∼11,500 cases. In Medvedeva, Vols & Wieling (2019), apply the approach proposed in Aletras et al. (2016) to 14 articles of this Convention to predict rulings of violation: Highest results in terms of F1-score metrics are 77% to predict whether articles will be violated, with a score of 79% in one experiment that used judges’ names alone. Moreover, in Aletras et al. (2016), Medvedeva, Vols & Wieling (2019), the authors use a data set which only includes decisions of the European Court of Human Rights written in English.

Further work also addresses legal judgment prediction from the Supreme People’s Court of China. For example, Li et al. (2019) use 1,367,654 cases from the Supreme People’s Court of China, extracting physiological characteristics and descriptions of the facts to predict specific law articles used in legal decisions, as well as charge and prison terms. The problem is then modeled by using attention neural network and word embeddings, with their approach attaining F1-scores of 0.41−0.96. Predictions of charges scored highest; prison term predictions the lowest. Hu et al. (2018) take advantage of Long Short-term Memory (LSTM) to build a model that uses facts and charges of legal cases. By using a criminal legal data set, this approach performs an F1-score of 73.1%. Moreover, Zhong et al. (2018) propose a multi-task learning framework for judgment prediction that uses topological dependencies among legal subtasks. When predicting charges, this framework is able to reach an F1-score of 78.3%. Last, Yang et al. (2019) propose a Multi-perspective Bi-feedback Network for multiple legal subtasks to also predict legal decisions from the Supreme People’s Court of China. The authors assessed this approach by predicting charges and it achieves an F1-score of 86.7%.

There are also other works from the literature that focus on various countries. In Ferreira Bertalan & Seron Ruiz (2020), the authors propose an approach to predicting Brazilian legal decisions for second-degree murder and active corruption crimes. The authors modeled the experiment as a binary prediction problem by using supervised machine learning algorithms on 782 cases from the São Paulo State higher court, selecting only findings of strict innocence or guilt. Among the algorithms assessed, the Regression Tree achieved the highest performance, with an F1-score of 98% for the active corruption data set, which contained 158 “innocent” and 31 “guilty” records. Regarding Unite Kingdom, Strickson & De La Iglesia (2020) address the prediction of legal case decisions from the United Kingdom and their best model achieved an F1-score of 69.02%. To predict case outcomes from the Supreme Court of the Philippines, Virtucio et al. (2018) propose an approach that reaches an accuracy of 59% by using Random Forest classifier and n-grams for feature extraction. By addressing the prediction of the Supreme Court of Thailand, Kowsrihawat, Vateekul & Boonkwan (2018) use Bi-GRU and attention model and have an F1-score of 66.67%. In the context of the Supreme Court of Turkey, Mumcuoglu et al. (2021) approach (Mumcuoglu et al., 2021) has an accuracy of 93.2% and an F1-score of 0.87 for tax cases. In Niklaus, Chalkidis & Stürmer (2021), the authors evaluate state-of-the-art BERT-based methods by using a multilingual *corpus* in German, French, and Italian with ∼85,000 records. The best results achieved an F1-macro score of 70% for German and French languages.

Additional related work applies machine learning techniques to other legal tasks. In Long et al. (2018), the authors propose a framework for automatically judging legal decisions by using attention-based neural network models, applying the approach to divorce decisions in China. In Shulayeva, Siddharthan & Wyner (2017), the authors separate legal principles from case facts within legal documents by using a Naive Bayes Multimodal classifier. The approach proposed in Elnaggar, Otto & Matthes (2018) uses transfer learning to recognize words which remain the same in various contexts, yet whose meaning changes, *i.e*., a named-entity linking task. Barros, Lorenzi & Wives (2018) use Bayesian networks to classify legal decisions from a Brazilian labor court and concluded that litigation success rates of employees and employers are approximately equal. In Ruhl, Katz & Bommarito (2017), the authors expose perspectives on how complex systems are useful for supporting policy-makers on legal-related topics such as appellate jurisprudence and tax policy analysis. Last, in Chouldechova (2017), Chouldechova shows that, although recidivism predictive instruments (RPIs) should use data-driven risk assessment techniques, these techniques might bias RPIs when the recidivism rate is not the same for different groups.

We are also motivated by industry results showing that intelligent systems can perform better than legal experts (https://www.bbc.com/news/technology-41829534). Our hypothesis is that *by taking advantage of Natural Language Processing (NLP) and Machine Learning techniques it is possible to build a system that makes high-quality legal decision predictions*. We propose an approach for predicting the outcome of Brazilian court rulings, in addition to predicting whether these decisions will be unanimous. Our proposal is different from Ashley & Brüninghaus (2009), Aletras et al. (2016), Katz, Bommarito & Blackman (2017), Medvedeva, Vols & Wieling (2019), Li et al. (2019), which address outcome prediction related to United States and European courts, and legal matters in China. Moreover, in contrast to Ashley & Brüninghaus (2009), Aletras et al. (2016), we trained a model on a thousand-scale data set containing 4,043 cases, and, in contrast to Aletras et al. (2016), Medvedeva, Vols & Wieling (2019), our approach does not rely only on binary classification nor requires that the case data set should be categorized by specific law articles.

The remainder of this paper is structured as follows. In “ Material and Methods”, we present details on the aforementioned problem such as the case study, the data set, and the methodology employed. “Results” presents the results while “ Conclusion” summarizes our investigation, provides our conclusions, and proposes future directions.

## Material and Methods

The research question which guides our study is *how to predict Brazilian court legal decisions with a satisfactory level of accuracy, as well as predict decision unanimity*. To better understand the addressed problem, we explain how Brazilian courts work in “Brazilian Legal System” and we then provide further information about the proposed methodology in “Material and Methods” which also describes the data set and the machine learning models.

### Brazilian legal system

This section introduces an overview of the Brazilian legal system in “A Generic Case Study” and explains how we labeled the cases in “Case Labels”.

#### A generic case study

By choosing Brazilian courts as a case study to validate our approach, we enable our contribution to be generic as Brazilian judge decisions share the same concepts and fundamentals of other law systems. We believe the Brazilian court system provides a useful context in which to validate our approach, given several key similarities it shares with other legal systems. In the United States of America, for instance, US Courts of Appeals are appellate courts that sit below the US Supreme Court. Appeals are heard in a panel of three judges and do not use a jury. Even though, differently from Brazilian courts, not all the court’s opinions are published (Wilson, 2003). The structure of those opinions is not very different from the decisions used in this study. The opinion starts with an overview of the case. It is followed by the history of the case, especially the procedure that was followed, the facts, and the statute that was applied. Then, the opinion states the standard of review that will apply, the actual analysis of the case followed by the conclusion (Wilson, 2003).

In France, the Appellate Court (*Cour d’appel*) also issues decisions based on a multi-judge panel. The decision (*Arrêt*) also has a standard structure, comprised of the legal basis for the appeal, case history, and the final decision (*dispositif*) (Française, 2019). We recall similarly, in Brazil, court decisions (*Acórdãos*) include Report (*Relatório*), Legal Principles (*Fundamentos*), Votes (*Dispositivo*), detailed Summary (*Ementa*), and further metadata such as judgment date, attorneys, prosecutors, and judges (do Brasil, 2015). Furthermore, our method may be suitable for several other legal systems whose decisions rely on more than one judge and whose published decision summary documents contain information about the case, and the explanations and decisions of individual judges. In other words, all the legal prediction tasks used in our methods can be applied to other legal systems as data collection, modeling, segmentation, and classification can be used similarly (c.f. “A Generic Case Study”).

Brazil is a Federation of States which have their own State Supreme Courts (*Tribunais de Justiça*). These Courts mainly hear appeals from the lower courts and are divided by subjects. For example, the State Supreme Court of Alagoas (*Tribunal de Justiça de Alagoas*) is divided between criminal and civil cases. Moreover, the State Supreme Court of Alagoas is composed of fifteen judges and there are three divisions of civil cases and one of criminal. Each division is composed of three judges and these subdivisions function as appellate courts. One of the fifteen judges of the State Supreme Court of Alagoas presides the Court and another is the vice-president, with one other judge functioning as an internal auditor. These judges have mainly administrative duties but they can take part in rulings of special cases that are judged by the court with all its judges. The judge that presides the court also has the important duty to admit appeals to The Brazilian Supreme Court (STF or *Supremo Tribunal Federal*).

When lodging an appeal with a State Supreme Court one should bring forward every argument to reform the original judgment from the lower court. Also, it is possible to lodge an appeal for just a part of the sentence. In this case, the upper court will only examine the part of the sentence that is the object of that appeal. Therefore, predicting case outcomes help attorneys to better prepare their appeal. Further, it is possible for a court to reform only part of the sentence in which case the appeal will be partially granted. When an appeal has been partially granted that means that the sentence has been reformed, not fully reformed, but reformed nonetheless. Hence, partially-granted appeals are legally closer to fully-granted appeals than to denied appeals.

#### Case labels

Regarding the process flow of the Brazilian appeals system, when lawyers lodge an appeal in a lower court, this appeal is submitted to an upper court and then analyzed by a panel of three judges to check whether the appeal can be decided by the upper court (do Brasil, 2015). If the appeal does not meet the formal requirements, the appeal is not accepted by the court and it is identified as not recognized (*não conhecido*) thus not judged beyond the formal requirements by the court. Otherwise, the appeal is judged and might fit into various categories. We, therefore, assumed that court decisions can be classified by using the following labels:

• “not recognized”, when the appeal was not accepted to be judged by the court;

• “yes”, for fully favourable decisions;

• “partial”, for partially favourable decisions;

• “no”, when the appeal was denied;

• “interrupted” (*prejudicada*), meaning the case could not be decided because of an impediment such as a party died or otherwise failed to pursue the case;

• “administrative”, when the decision is based on a matter related to court administration, *e.g*., conflict of competence between lower court judges.

In addition to the decision outcome labels listed above, a related aspect is decision unanimity, meaning that regardless of outcome type, the decision itself can be unanimous or not, as:

• “unanimous” which means that the decision was unanimous among the three judges that voted in the case;

• “not-unanimous”, meaning that the decision of at least one of the judges differed from that of the others.

The importance of knowing if a decision of a court is unanimous or if there is dissent usually lies especially in hard cases as dissent can represent a tendency of the court to overrule its former rulings. In Brazilian upper State courts, easy cases are often subject to unanimous decisions and unanimous decisions are prevalent. Hence, if we can predict that a decision will be unanimous or not we may be spotting a hard case in advance or tracking a tendency to overrule.

### Methodology

In this section, we present our assumptions and we detail the legal prediction tasks. We also provide information on the data set and the models used by our methodology.

#### Legal prediction tasks

Our approach shares the same assumption of Aletras et al. (2016): “there is enough similarity between (at least) certain chunks of the text of published judgments and applications lodged with the Court and/or briefs submitted by parties concerning pending cases”. In other words, we assume that the part of a legal decision that describes the case has enough similarities to the way attorneys lodge an appeal. Even though considering that people have different writing styles, our assumption is very reasonable since legal text styles tend to not differ substantially when they address related concerns. Hence, the case descriptions that we used in this paper were extracted from legal decisions.

**Figure 1:**
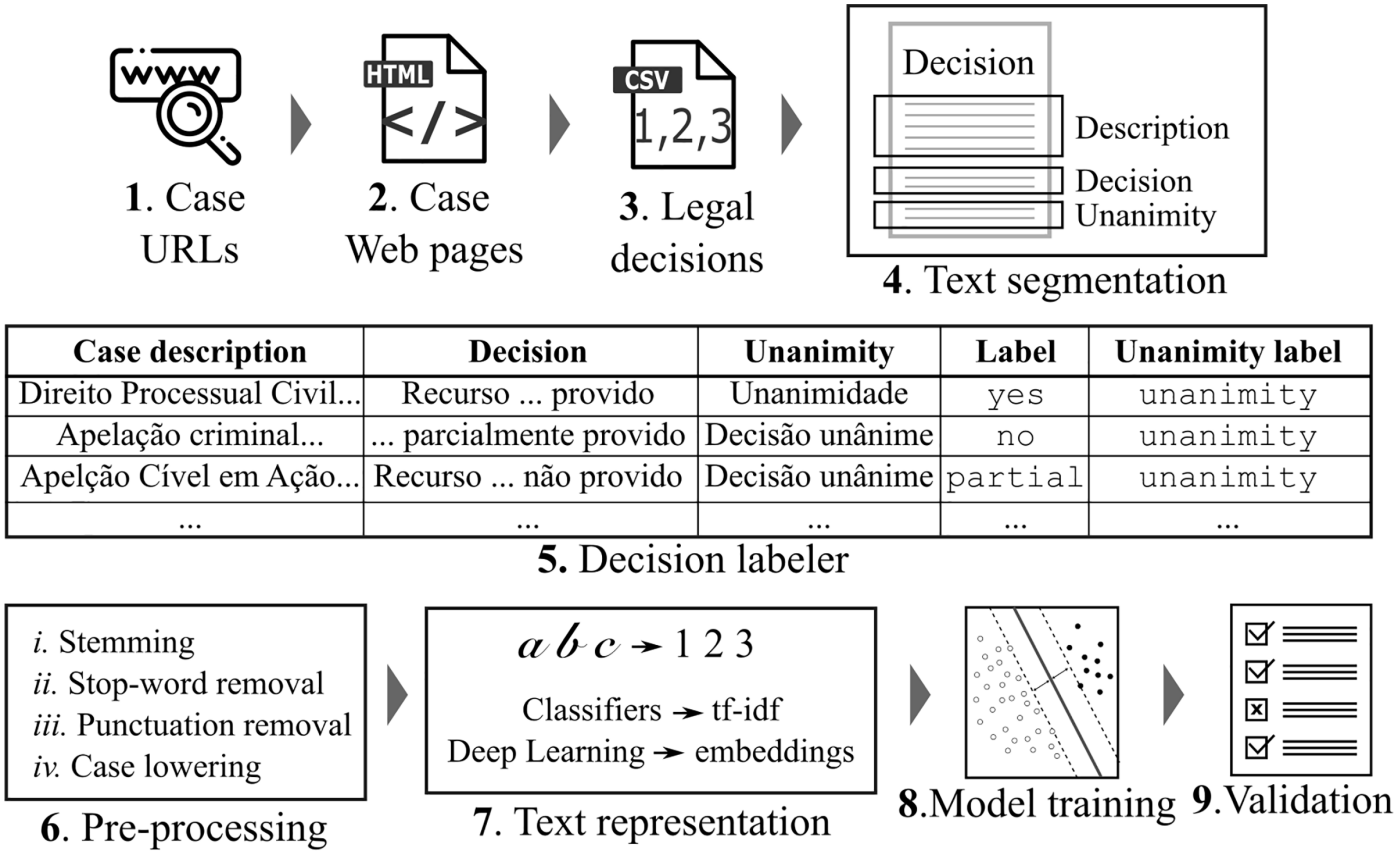
Methodology. First, legal case data are collected from the Web and segmented into sections.Then, we label the cases according to their decision and unanimity aspects. As follows, we pre-processthe case descriptions and represent them in number vectors. Last, we train different machine learningalgorithms and evaluate the trained models.

Figure 1 depicts an overview of our approach along with its data life-cycle. Because legal data sets are often available as PDF or HTML files at Web sites, it is very hard to extract legal decision data from them. The great CAIL Chinese data set (Xiao et al., 2018) indeed is the exception, with ∼2.6 million law cases. In Brazil, each court defines the technology it will use; therefore, there are different user interfaces for downloading legal case documents. Moreover, some Brazilian courts’ Web sites are protected by CAPTCHA (CAPTCHA is an acronym for Completely Automated Public Turing test to tell Computers and Humans Apart) which makes it harder to build Web scrapers for instance.

We developed a **Web scraper** for collecting data from Brazilian courts. The scraper first searched for the URL that contains the list of court cases (c.f. Step 1 in Fig. 1). Then, the scraper extracted from these HTML files the specific case URLs and downloaded their data (c.f. Step 2 in Fig. 1). Next, it extracted the metadata and the contents of legal cases and stored them in a CSV file format (c.f. Step 3 in Fig. 1).

We then performed a **text segmentation** task (c.f. Step 4 in Fig. 1) to identify target sentences within the legal decisions, containing information about the case description, the decision, and the decision’s unanimous or non-unanimous nature. Next, we extracted the features by assigning labels (c.f. “Case Labels”) and labeling the target sentences accordingly (c.f. **decision classification** task in Step 5 in Fig. 1). Tables 1 and 2 show three records of the classified data. In Table 1, there are texts that refer to the case description (*Case description* column), texts which contain decisions (*Decision* column), and text indicating the decision’s unanimity status (*Unanimity* column). In Table 2, the *Decision* column texts from Table 1 are classified according to decision outcome (“yes”, “no”, and “partial”) and Table 1 Unanimity column texts were classified as “unanimous” or “non-unanimous”. For example, we observe the Record 1 sentence from Table 1 is assigned a decision label of “yes” and a unanimity label of “unanimous” in Table 2.

**Table 1:** The data set includes the texts that describe the case description, the decision, and the decision unanimity.

Data	Case description	Decision	Unanimity
**Record 1**	Direito Processual Civil…	Recurso conhecido e provido	Unanimidade
**Record 2**	Apelação criminal…	Recurso conhecido e parcialmente provido	Decisão unânime
**Record 3**	Apelação Cível em Ação…	Recurso conhecido e não provido	Decisão unânime

**Table 2:** Data set classification. *E.g*., in Record 1, *provido* means a favorable (“yes”) decision and Unanimidade was classified as a “unanimous” decision.

Data	Label	Unanimity label
**Record 1**	“yes”	“unanimous”
**Record 2**	“patial”	“unanimous”
**Record 3**	“no”	“unanimous”

For the **Pre-processing** (c.f. Step 6 in Fig. 1), we used the following techniques to improve word representation and improve modeling efficiency: word stemming, removal of stop-words and punctuation, and lower-casing. Word stemming is useful to model semantically similar words as the same words. Stop-words–*e.g*., *and, or, not, at*—and punctuation are not meaningfully important and were therefore removed. Last, we lower-cased all initial capital letters to make a word recognizable to the system as the same despite whether its letter was originally in upper or lower case.

Concerning text representation into vectors, our methodology takes advantage of different approaches. We used the Term Frequency-inverse Document Frequency (tf-idf) statistics to numerically represent the text as unigrams for the supervised models. Tf-idf increases the importance of relevant words while decreasing that of words that appear frequently but are nonetheless irrelevant. For the Deep Learning models, we use word embeddings Le & Mikolov (2014) which represent texts as fixed-length feature vectors whose features were generated by using unsupervised learning algorithms.

After language modeling, Steps 8 and 9 (c.f. in Fig. 1) refer to model training and validation. We used part of the data set to train various machine learning models (c.f. “Models”) which are then assessed by using the rest of it. For training case outcome models, the input data is the tuple <*Case description*, *Label*>, c.f. Tables 1 and 2 respectively, while unanimity training models use the tuple <*Case description*, *Unanimity Label*>.

**Figure 2:**
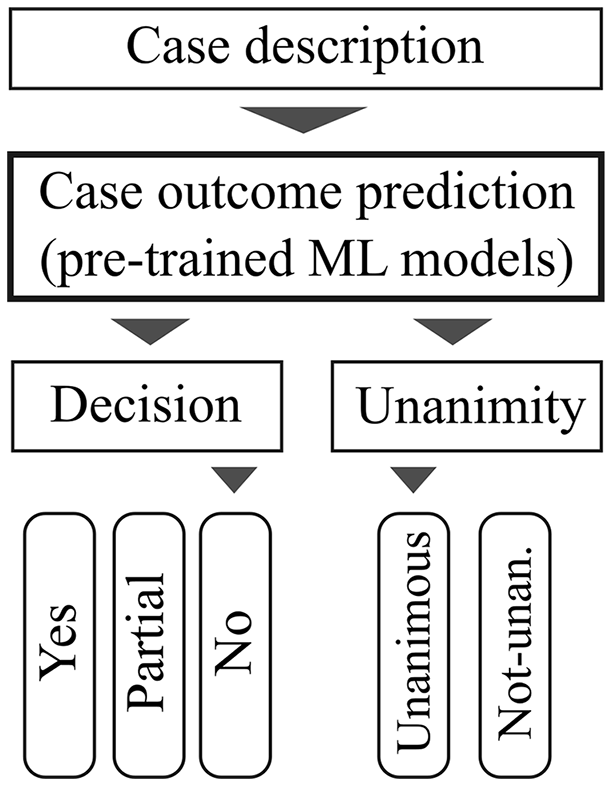
The user interface only requires case description and the Machine Learning models predict thecase outcome and its unanimity.

Figure 2 illustrates our approach’s practicality and usability. First, the user provides the case description as input. This case description is forwarded to a case outcome prediction engine that uses previously trained models. The prediction results are therefore the most appropriate results which represent the case description. There are two predictive results: one according to the case outcome and another which predicts whether this case outcome will be unanimous. In Fig. 2, the predictions are that the court would reject the appeal unanimously. This design provides an intuitive interface for end-users such as attorneys, prosecutors, and even judges who need to better understand their jurisprudence.

#### Data

We used the Web scraper (c.f. Fig. 1) to download legal cases from a Brazilian State higher court (appellate court), the *Tribunal de Justiça de Alagoas*. The total amount of scraped data is 2 GB which holds 4,762 Ementa legal decisions. Then, we removed duplicate decisions based on similar case descriptions to avoid biasing the data, yielding 4,332 records. Repeated case descriptions occur owing to very similar cases that share descriptions. For the sake of predictability, we removed all the decisions classified as “interrupted”, not “recognized”, and “administrative”, as these labels refer to unusual situations not useful for the prediction purposes addressed by this paper (c.f. “Case Labels”). Thus, we only used legal decisions labeled as “yes”, “no”, and “partial”, yielding a data set of 4,043.

**Figure 3:**
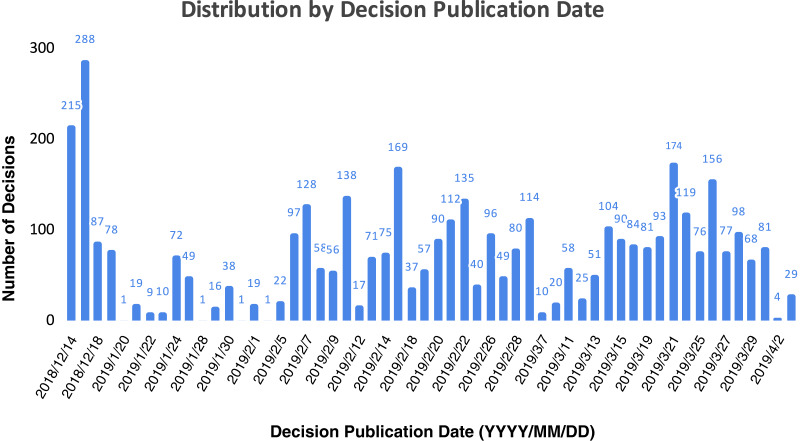
Data set distribution by decision publication date.

Regarding the dates on which the decisions were published, Fig. 3 depicts the distribution of the 4,043 legal-decision data set per month. We used the decision publication date to plot the data set distribution as this is when a decision can affect a third party. The whole date range is 110 days: while the publication date of the first decision is December 14th, 2018, and the last publication date is April 3rd, 2019. Concerning the data distribution, the great number of published decisions in December is common in Brazil as the Brazilian legal system has a yearly deadline in 19th December, hence it usually has high rates of legal decision publications in this month. On the other months, it is also expected to have irregular decision publication dates owing to repetitive mass cases which are judged in a bundle, therefore some decision publication dates are higher than others. With respect to further data set statistics regarding the number of words in each sample, the mean is 119, the median is 88, the lowest sample has 12 words, and the biggest sample has 1,400 words. Moreover, the vocabulary size is 17,341.

#### Models

Our methodology uses for prediction tasks classifiers and other models that take advantage of artificial neural networks. The advantage of the classic machine learning algorithms–such as classifiers–is that they perform better with less data than the state-of-the-art Deep Learning models. Also, classifiers are far more interpretable and their training time is lower, in comparison with deep learning models. Deep learning models, on the other hand, try to learn high-level features from data in an incremental manner. This eliminates the need for domain expertise and hard-core feature extraction. Furthermore, in training our models, we identify the most suitable parameters to predict decisions based on the training data set, undertaking two Natural Language Processing tasks, to make predictions regarding both decision outcome and unanimity, based on the case description.

Regarding the classifiers, our methodology uses Gaussian Naive Bayes (GNB), Decision Tree (DT), Support-vector Machine (SVM), Random Forest (RF), eXtreme Gradient Boosting (XGBoost). The **Gaussian Naive Bayes (GNB)** is a probabilistic model which relies on Bayes’ theorem (Rish, 2001) and assumes a Gaussian likelihood for the features. It is said to be naive since it assumes independence between the features. The **Decision Tree (DT)** is a simple and comprehensible algorithm that uses a tree data structure to create decision paths to how the data will be classified. The great advantage of the Decision-tree model is its simplicity and its ability to know the rules used to classify each class (Su & Zhang, 2006). The **Support-vector Machine (SVM)** (Cortes & Vapnik, 1995) is a supervised machine learning model that has been successfully used to solve several pattern recognition problems such as Burges (1998). The SVM model aims at maximizing the separation among classes by taking into account a margin.

The **Random Forest (RF)** model combines ensemble learning with decision trees. This is also a simple algorithm and suitable for high-dimensional data such as text classification for instance (Xu et al., 2012). Last, the **eXtreme Gradient Boosting (XGBoost)** is an implementation of the general gradient descent “boosting” paradigm (Friedman, 2001). XGBoost is a scalable and accurate implementation of gradient boosting machines and it has proven to push the limits of computing power for boosted trees algorithms as it was built and developed for the sole purpose of model performance and computational speed. Usually, ensemble approaches outperform single models in terms of performance measures.

Moreover, we use the following high-performing deep learning models: Bidirectional Encoder Representations from Transformers (BERT), Long Short-Term Memory (LSTM), Gated Recurrent Unit (GRU), Bidirectional Long Short-Term Memory (BiLSTM), and Convolutional Neural Networks (CNN). **Bidirectional Encoder Representations from Transformers (BERT)** is a transformer-based machine learning technique for natural language processing pre-training developed by Google. BERT-Imbau is a version of BERT that is pre-trained for Brazilian Portuguese (Souza & Lotufo, 2020). **Long Short-term Memory (LSTM)** is an artificial Recurrent Neural Network (RNN) architecture used in time series forecasting and natural language processing (Hochreiter & Schmidhuber, 1997). Unlike standard feed-forward neural networks, LSTM has feedback connections. Thus, it can process not only single data points (*e.g*., images) but also entire sequences of data such as speech or video.

**Gated recurrent units (GRUs)** are a gating mechanism in recurrent neural networks, introduced in 2014 by Kyunghyun Cho et al. (2014). The GRU is like a long short-term memory (LSTM) with a forget gate; however, it has fewer parameters than LSTM as LTSMs do not have an output gate. In deep learning, a **Convolutional Neural Network (CNN, or ConvNet)** is a class of artificial neural networks, most commonly applied to analyze visual imagery and natural language processing. CNNs are also known as shift invariant or space invariant artificial neural networks (SIANN) and are based on the shared-weight architecture of the convolution kernels or filters. these kernels slide along input features and provide translation equivariant responses known as feature maps.

## Evaluation

This section explains how we evaluated our approach. In “Experimental Setup”, we describe the metrics, the execution environment, the model parameters, and the implementation. Then, in “Results”, we present the evaluation scenarios and discuss the results.

### Experimental setup

We used the data set introduced in “Data” which has 4,043 legal decisions from the State Supreme Court of Alagoas (Brazil). Regarding the evaluation metrics, we used the F1-score (macro), Precision, Recall, and Accuracy metrics. Concerning the data split criterion, we did not use the decision publication date as jurisprudence takes time to change, therefore one would not find any significant change in our data set date range (c.f. Fig. 3). Therefore, we performed the *n*-fold cross-validation approach. Cross validation is one of the state-of-the-art methods to test Machine Learning models for Natural Language Processing tasks (Fong & Holmes, 2020). This method divides the data set in *n* slices randomly chosen then it uses one slice to test the models and the other slices as training data set. The validation is executed *n* times and then we calculate the mean of the *n* measured metrics. In this experiment, we set *n* = 5, thus training data sets had 80% of the total data set while test data set had 20%. Last,

Moreover, we used two computers to execute the experiments: one with 40 cores, Intel Xeon CPU E5-2687W v3 @ 3.10 GHz, 128 GB main memory, and SSD for the supervised learning models. We used another computer to run the Deep Learning experiments whose characteristics are: 12 cores, Intel Core CPU i7-8700K @ 3.70 GHzIntel, 32 GB main memory, SSD, and a Graphics Processing Unit (GPU) Nvidia Geforce 1080ti.

Regarding model hyperparameters, we used the grid search technique to optimize the model parameter values based on specific value ranges. The parameters for the Decision Tree model were the following: *minimum number of records from 10 to 100 in steps of 10, max depth of the tree from 1 to 20 in steps of 2*. The parameters of the SVM were: *Cs = [0.001, 0.01, 0.1, 1, 10], gammas = (0.001;0.01;0.1*; Abadi et al., 2015), *degree = [1,2,3,4,5,6,7,8,9] and kernel = [ ’linear’, ’poly’, ’rbf’, ’sigmoid’, ’precomputed’]*. The parameters of XGBoost were: *learning rate = [0.1, 0.2, 0.3], maximum depth from 1 to 6, minimum child weight from 1 to 5, and the number of estimators from 100 to 1000 in steps of 100*. The parameters of Random Forest were the following: *number of estimators from 100 to 1000 in steps of 100 and max depth from 1 to 6*. Moreover, there is no parameter specifications for the Gaussian Naive Bayes model since it is inherently not able to be tuned. With respect to BERT-Imbau model, we configured the variables as following: *Max length = 200, Train Batch Size = 8, Test Batch Size = 8, Epochs = 5 and, Learning Rate = 1e-05*. Last, regarding LSTM, BiLSTM, GRU, and CNN, the tuning variables are described next: *Max Length = 100, Train Batch Size = 16, Test Batch Size = 16, Epochs = 20, and Max Features = 8000*. We searched the number of neurons in the hidden layer from 50 to 500 in increments of 50 and the learning rate = (0.01;0.001;0.0001;0.00001).

On implementation details, we used generic machine learning algorithms and conventional software tools to build and process the data pipeline to make our approach easily extensible. We developed a prototype in Python which is able to support various languages and other legal systems. We used the Natural Language Toolkit (NLTK) framework (Loper & Bird, 2002) for Natural Language Processing in such a way that our prototype is easily configurable for various languages in addition to Portuguese. Moreover, we used Scikitlearn (Pedregosa et al., 2011), PyTorch (Paszke et al., 2019), and TensorFlow (Abadi et al., 2015) which implement the prototype’s machine learning algorithms in Python. The prototype also provides an interface that can be accessed from Web browsers. Last, reproducible materials are available at GitHub (https://github.com/proflage/predicting-brazilian-court-decisions).

### Results

We divided the results into five scenarios. Scenarios 1, 2, and 3 address decision outcome prediction while Scenarios 4 and 5 address unanimity prediction. We provide further details on the Scenarios and their results next.

#### Scenarios 1, 2, and 3

**Scenarios 1, 2,** and **3** aim at investigating how the models perform when predicting case outcomes for different data distributions. Thus, Scenarios 1, 2, and 3 are distinguished by the distribution of each label among the data set, as Table 3 depicts. Scenario 1 uses the whole data set. The sum of its “no”, “partial”, and “yes” records is 4,043. In order to perform a predictive analysis over a more uniformly distributed data set, we randomly removed 1,549 “no”-labeled decisions to have the same number of “partial”-labeled decisions, thereby creating Scenario 2. Finally, in Scenario 3, we also evaluated our approach by reducing the prediction problem to a binary case outcome forecast, reclassifying all “partial”-labeled decisions as “yes”. This is based on the assumption that partially favorable decisions are closer to favorable decisions rather than non-favorable decisions as both “partial” and “yes” decisions of the lower court were reverted.

**Table 3:** Number of data set records according to their decision labels for Scenarios 1, 2, and 3. These scenarios perform case outcome predictions.

Scenarios	“no”	“partial”	“yes”
**1**	2,415	866	762
**2**	866	866	762
**3**	2,415	–	1,628

Tables 4 and 5 depicts the results of **Scenario 1** and **Scenario 2** respectively. The best performance for F1-score (macro) was achieved by the Bert-Imbau model with ∼73% for Scenario 1 and XGBoost obtained ∼71% for Scenario 2. In terms of the other performance measures, XGBoost outperformed all the other models in **Scenario 1** and **Scenario 2** regarding the other three performance metrics (Precision, Recall, and Accuracy). We expected this as the XGBoost model is state-of-the-art for several tasks. XGBoost usually performs better than other models for several tasks as ensemble models add diversity to the trained model. Moreover, we also expected good performance from the SVM model, however, it surprised us by achieving higher results than expected: the SVM model achieved close results comparing to the XGBoost model for all metrics. Last, the classic machine learning models were outperformed by BERT, LSTM and GRU. BERT obtained comparable results to SVM because we used BERT-Imbau, a BERT pre-trained version for Portuguese.

**Table 4:** Results of Scenario 1: case outcome predictive analyses. Mean and standard deviation of F1-score, Precision, Recall, and Accuracy metrics over a five-fold validation.

Models	F1-score	Precision	Recall	Accuracy
Gaussian NB	0.4772 *±* 0.0245	0.4746 *±* 0.0229	0.4836 *±* 0.0264	0.5451 *±* 0.0220
Decision Tree	0.6260 *±* 0.0176	0.6652 *±* **0.0095**	0.6064 *±* 0.0200	0.7203 *±* 0.0120
SVM	0.6838 *±* 0.0207	0.7204 *±* 0.0185	0.6620 *±* 0.0212	0.7601 *±* 0.0137
Random Forest	0.2948 *±* **0.0038**	0.5244 *±* 0.0139	0.3564 *±* **0.0021**	0.6122 *±* **0.0013**
XGBoost	0.7015 *±* 0.0191	**0.7308** *±* 0.0212	**0.6827** *±* 0.0181	**0.7702** *±* 0.0142
BERT-Imbau	**0.7342** *±* 0.0632	0.6609 *±* 0.1602	0.6301 *±* 0.1297	0.7342 *±* 0.0632
LSTM	0.7105 *±* 0.0151	0.6444 *±* 0.0251	0.6102 *±* 0.0222	0.7105 *±* 0.0151
GRU	0.7175 *±* 0.0194	0.6623 *±* 0.0283	0.6082 *±* 0.0366	0.7175 *±* 0.0194
BiLSTM	0.5549 *±* 0.0764	0.4343 *±* 0.1036	0.4127 *±* 0.0883	0.5549 *±* 0.0764
CNN	0.6071 *±* 0.0198	0.6529 *±* 0.0281	0.5898 *±* 0.0209	0.7032 *±* 0.0153

**Note:**

Bold numbers represent the best results.

**Table 5:** Results of Scenario 2: case outcome predictive analyses. Mean and standard deviation of F1-score, Precision, Recall, and Accuracy metrics over a 5-fold validation.

Models	F1-score	Precision	Recall	Accuracy
Gaussian NB	0.5458 *±* 0.0183	0.4746 *±* 0.0229	0.5469 *±* 0.0186	0.5457 *±* 0.0179
Decision Tree	0.6315 *±* **0.0160**	0.6386 *±* **0.0134**	0.6344 *±* **0.0161**	0.6323 *±* **0.0166**
SVM	0.6972 *±* 0.0228	0.6997 *±* 0.0224	0.6985 *±* 0.0221	0.6977 *±* 0.0227
Random Forest	0.6064 *±* 0.0340	0.6511 *±* 0.0343	0.6148 *±* 0.0313	0.6251 *±* 0.0309
XGBoost	**0.7154** *±* 0.0262	**0.7163** *±* 0.0265	**0.7154** *±* 0.0261	**0.7161** *±* 0.0265
BERT-Imbau	0.6278 *±* 0.1206	0.6211 *±* 0.1828	0.6297 *±* 0.1210	0.6278 *±* 0.1206
LSTM	0.6032 *±* 0.0309	0.6114 *±* 0.0279	0.6021 *±* 0.0308	0.6032 *±* 0.0309
GRU	0.6137 *±* 0.0306	0.6198 *±* 0.0298	0.6134 *±* 0.0302	0.6137 *±* 0.0306
BiLSTM	0.4356 *±* 0.0738	0.4355 *±* 0.0789	0.4340 *±* 0.0739	0.4356 *±* 0.0738
CNN	0.6177 *±* 0.0219	0.6309 *±* 0.0205	0.6183 *±* 0.0212	0.6177 *±* 0.0219

**Note:**

Bold numbers represent the best results.

Further, our assumption about Scenario 1 was that its data set would strongly bias the model and, eventually, achieve higher performance than Scenario 2. Nevertheless, we observe only a slight model performance increase in Scenario 1 in comparison to Scenario 2 (c.f. Tables 4 and 5, columns *F1-score, Precision*, and *Accuracy*). This happened because F1-score is a geometric mean between Precision and Recall metrics as well as F1-score is more affected by False Negatives (the majority class). Last, Scenario 1 performed worse than Scenario 2 only in terms of Recall as expected. In terms of accuracy, the best model was XGBoost, followed by the results of the SVM. Furthermore, BERT-Imbau was the third-best model regarding Accuracy.

In **Scenario 3** (c.f. Table 6), the most accurate results were achieved by the XGBoost model, with ∼80% F1-score and ∼81% Accuracy. The second model with the best results was SVM, obtaining ∼79% F1-score. Moreover, Scenarios 1, 2, and 3 presented low standard deviation values, which confirms our assumption that the 5-fold cross validation was appropriate. Similar to Scenarios 1 and 2, the GRU and BiLSTM models did not perform as well as the classic machine learning models since these models usually perform better for huge data sets.

**Table 6:** Results of Scenario 3: case outcome predictive analyses. Mean and standard deviation of F1-score, Precision, Recall, and Accuracy metrics over a 5-fold validation.

Models	F1-score	Precision	Recall	Accuracy
Gaussian NB	0.6028 *±* 0.0153	0.4746 *±* 0.0229	0.6142 *±* 0.0135	0.6055 *±* 0.0171
Decision Tree	0.7495 *±* **0.0109**	0.7516 *±* **0.0118**	0.7480 *±* **0.0104**	0.7606 *±* **0.0110**
SVM	0.7911 *±* 0.0145	0.8057 *±* 0.0131	0.7846 *±* 0.0148	0.8051 *±* 0.0128
Random Forest	0.4874 *±* 0.0609	0.7512 *±* 0.0724	0.5568 *±* 0.0339	0.6419 *±* 0.0279
XGBoost	**0.8022** *±* 0.0131	**0.8108** *±* 0.0130	**0.7974** *±* 0.0131	**0.8135** *±* 0.0121
BERT-Imbau	0.7830 *±* 0.0684	0.7636 *±* 0.1448	0.7578 *±* 0.0925	0.7830 *±* 0.0684
LSTM	0.7706 *±* 0.0171	0.7636 *±* 0.0177	0.7562 *±* 0.0189	0.7706 *±* 0.0171
GRU	0.5973 *±* 0.0004	0.3186 *±* 0.1001	0.5000 *±* 0.0003	0.5973 *±* 0.0004
BiLSTM	0.5929 *±* 0.0765	0.5653 *±* 0.0861	0.5608 *±* 0.0830	0.5929 *±* 0.0765
CNN	0.7490 *±* 0.0225	0.7418 *±* 0.0230	0.7316 *±* 0.0280	0.7490 *±* 0.0225

**Note:**

Bold numbers represent the best results.

**Figure 4:**
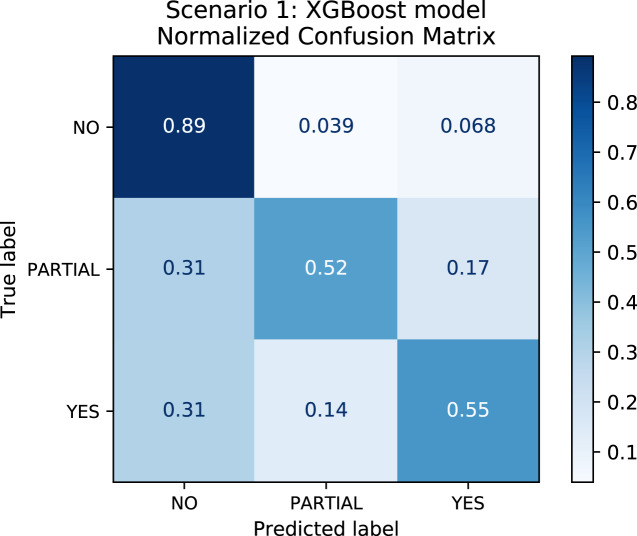
Confusion Matrix: Scenario 1 for the XGBoost model.

**Figure 5:**
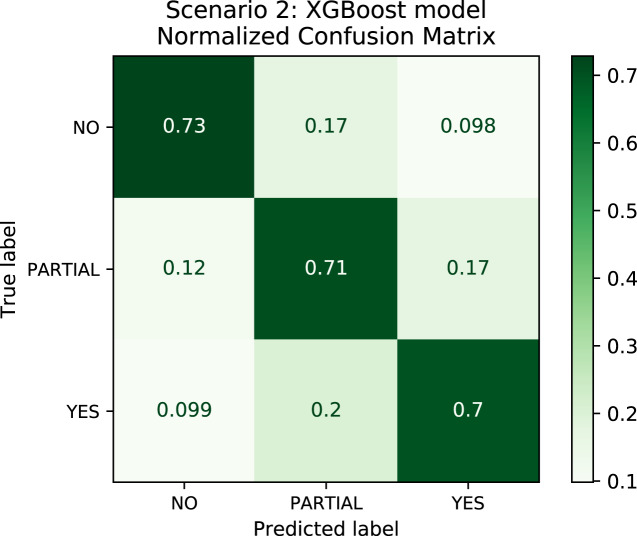
Confusion Matrix: Scenario 2 for the XGBoost model.

**Figure 6:**
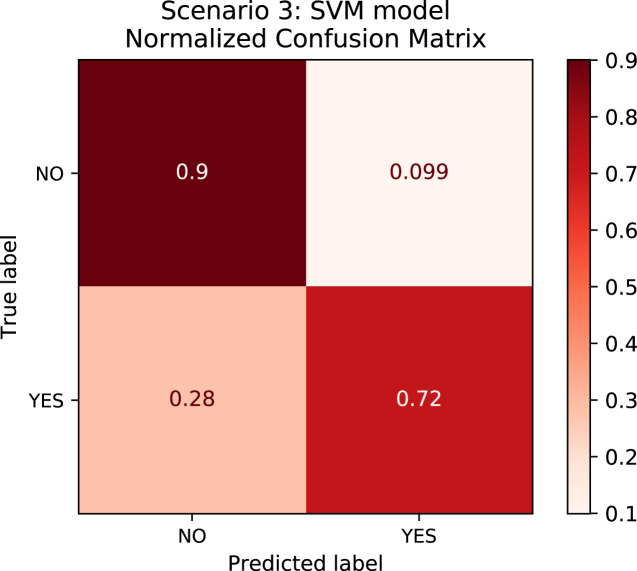
Confusion Matrix: Scenario 3 for the SVM model.

In order to better understand the relationship between the results and the bias towards the majority class, we plotted the confusion matrix for Scenarios 1 and 2 for one experimental run. We chose the XGBoost model only as it was the model that best performed in both scenarios in general. In Fig. 4, the model is biased towards the majority label “no” as expected since there are more records labeled no. Actually, this was the motivation to create Scenario 2: we assumed that uniformly distributing the data set would not bias the models. To check this assumption, we plotted the confusion matrix for Scenario 2 in Fig. 5. In Fig. 5, we observe that when the model did not correctly predict the label “partial”, in most of the cases it predicted “partial” as “yes” and vice-versa. Nevertheless, the model did not bias towards the label “no” in this scenario. Further, Fig. 6 depicts the confusion matrix for Scenario 3. We expected that the model would be biased to the label “no” as happened since there are more records labeled “no”.

#### Scenarios 4 and 5

The goal of **Scenarios 4** and **5** is to investigate how the machine learning models perform when predicting whether the three judges that compose the panel will be unanimous on the legal decision. In Table 7, Scenarios 4 and 5 are data sets representing the number of decisions with labels identifying unanimity behavior. To yield the 2,274 cases used in this assessment, we started with a data set of 4,332 where duplicate case descriptions had been removed (c.f. “Data”). We then removed records where either the decision itself did not contain any information about unanimity or our classifier did not manage to label, resulting in a data set of 2,289. Next, we removed decisions labeled as “interrupted”, “not-cognized”, and “administrative”–since, as explained, these labels are not relevant to our problem–resulting in a data set of 2,274.

**Table 7:** Distribution of data set records for Scenarios 4 and 5, which predicts judge unanimous behavior.

Scenarios	“not-unanimous”	“unanimous”
**4**	45	2,229
**5**	45	45

**Figure 7:**
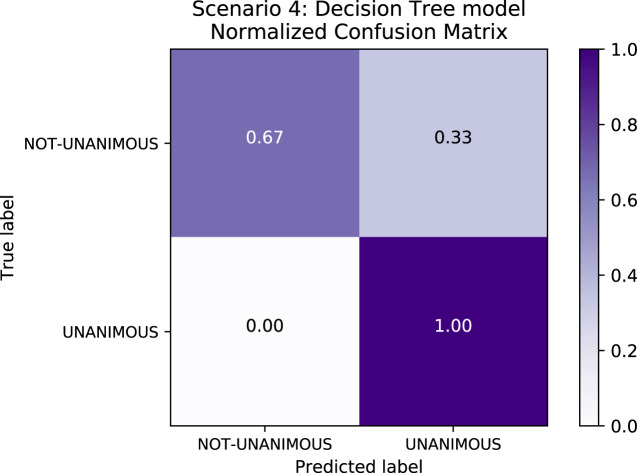
Confusion Matrix: Scenario 4 for the Decision Tree model.

Table 8 depicts the results of Scenario 4. The unanimous predictive results an F1-score of∼98% in **Scenario 4** are explained by the fact that, as expected, most of the decisions in the data set were unanimous–indeed, reflecting a trend in the population familiar to legal experts–and so all models exhibited bias relative to this label. Figure 7 depicts the confusion matrix for Scenario 4 and confirms the bias towards the “unanimous” label.

**Table 8:** Results of Scenario 4: prediction on decision unanimity. Mean and standard deviation ofF1-score, Precision, Recall, and Accuracy metrics over a 5-fold validation.

Models	F1-score	Precision	Recall	Accuracy
Gaussian NB	0.6425 *±* 0.0662	0.7322 *±* 0.0573	0.6088 *±* 0.0606	0.9802 *±* 0.0024
Decision Tree	0.8091 *±* 0.0579	0.8794 *±* 0.0466	0.7651 *±* 0.0649	**0.9877** *±* 0.0030
SVM	0.6718 *±* 0.0775	0.8475 *±* 0.0961	0.6213 *±* 0.0648	0.9833 *±* 0.0026
Random Forest	0.4950 *±* **0.0000**	0.4901 *±* **0.0000**	0.5000 *±* **0.0000**	0.9802 *±* **0.0000**
XGBoost	0.8065 *±* 0.0919	**0.9248** *±* 0.0994	**0.7437** *±* 0.0839	0.9885 *±* 0.0048
BERT-Imbau	0.9790 *±* 0.0043	0.5920 *±* 0.1900	0.5277 *±* 0.0556	0.9790 *±* 0.0043
LSTM	0.9854 *±* 0.0036	0.8884 *±* 0.1654	0.6550 *±* 0.0919	0.9854 *±* 0.0036
GRU	**0.9855** *±* 0.0039	0.8379 *±* 0.2056	0.6529 *±* 0.1053	0.9855 *±* 0.0039
BiLSTM	0.9852 *±* 0.0043	0.8024 *±* 0.2074	0.6549 *±* 0.1143	0.9852 *±* 0.0043
CNN	0.9854 *±* 0.0027	0.8822 *±* 0.1146	0.6921 *±* 0.0796	0.9854 *±* 0.0027

**Note:**

Bold numbers represent the best results.

To understand how our approach would perform when predicting unanimity by using a more uniformly distributed data set, we created **Scenario 5**. In this Scenario, we randomly removed “unanimous” labeled decisions to equal the number of “not-unanimous” decisions, resulting in a data set of 90 records total. The results of Scenario 5 (c.f. Table 9) performed satisfactorily in general as most models reached an F1-score of more than 78%, *e.g.*, the Random Forest model performed ∼*84%. Regarding the performance of the deep learning models, their poor performance is again owing to the size of the data set: deep learning models tend to highly perform for larger data sets*.

**Table 9:** Results of Scenario 5: prediction on decision unanimity. Mean and standard deviation of F1-score, Precision, Recall, and Accuracy metrics over a 5-fold validation.

Models	F1-score	Precision	Recall	Accuracy
Gaussian NB	0.6670 *±* **0.0288**	0.4746 *±* **0.0229**	0.6889 *±* **0.0272**	0.6889 *±* **0.0272**
Decision Tree	0.7863 *±* 0.0960	0.8051 *±* 0.0993	0.7889 *±* 0.0956	0.7889 *±* 0.0956
SVM	0.8206 *±* 0.0836	0.8289 *±* 0.0771	0.8222 *±* 0.0816	0.8222 *±* 0.0816
Random Forest	**0.8410** *±* 0.0576	**0.8691** *±* 0.0448	**0.8444** *±* 0.0544	**0.8444** *±* 0.0544
XGBoost	0.7648 *±* 0.0964	0.7735 *±* 0.0957	0.7666 *±* 0.0955	0.7666 *±* 0.0955
BERT-Imbau	0.6787 *±* 0.1043	0.7246 *±* 0.1039	0.6911 *±* 0.0963	0.6911 *±* 0.0963
LSTM	0.6577 *±* 0.1141	0.6552 *±* 0.1638	0.6577 *±* 0.1141	0.6577 *±* 0.1141
GRU	0.6866 *±* 0.1271	0.7097 *±* 0.1319	0.6866 *±* 0.1271	0.6866 *±* 0.1271
BiLSTM	0.7200 *±* 0.1045	0.7538 *±* 0.1159	0.7200 *±* 0.1045	0.7200 *±* 0.1045
CNN	0.7533 *±* 0.0483	0.7833 *±* 0.0538	0.7533 *±* 0.0483	0.7533 *±* 0.0483

**Note:**

Bold numbers represent the best results.

**Figure 8:**
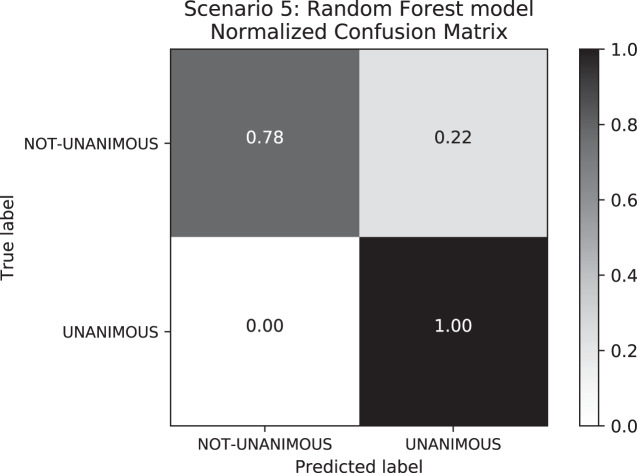
Confusion Matrix: Scenario 5 for the Random Forest model.

When comparing the differences between Scenarios 4 and 5, Scenario 4 has an F1-score higher than Scenario 5 owing to higher Recall values in Scenario 5. This happened because the false-negative rate–*i.e*., the labels that were supposed to be “not-unanimous” but were predicted as “unanimous”–is lower in Scenario 4 (c.f. Fig. 8). With respect to standard deviation, both Scenarios 4 and 5 presented low values; however, Scenario 5 reflected higher values than Scenario 4. Furthermore, the great difference between “unanimous” and “non-unanimous” decisions in Scenario 4 data set is surprising. We expected most of the decisions to be unanimous, however, not so high as the results showed for a Brazilian State Supreme Court.

## Conclusion

This paper proposes a methodology for predicting Brazilian court legal decisions that reaches an F1-score of 80.2% when employed for a Brazilian court data set with 4,043 cases. To our knowledge (This paper was first published as a Technical Report on April 20th, 2019 at the following address: https://arxiv.org/abs/1905.10348), this is the first study to predict Brazilian legal decisions. In addition to considering a binary predictive problem, *i.e.*, “no” and “yes” predictive results, our approach is also able to predict case outcomes by also predicting “partial” favorable decisions. In this context, our approach’s performance is an F1-score of 73.4%. The proposed method also predicts whether the decision will be unanimous, which applies to not only the Brazilian legal system but also several others whose decisions are adjudicated by more than one judge. The unanimity prediction performance of our approach is an F1-score of 84.1%. With respect to the Machine Learning models, we used various supervised classification algorithms and state-of-the-art Deep Learning models. The Deep Learning models, *i.e.*, BERT-Imbau, LSTM, GRU, BiLSTM, and CNN, were outperformed by classic machine learning models. The only exception was BERT when predicting a three-label case outcome for an imbalanced data set: BERT-Imbau reached an F1-score of 73.4% against 70.1% from XGBoost. Moreover, BERT-Imbau model obtained comparable results to XGBoost since BERT-Imbau is a BERT pre-trained model by using Portuguese *corpus*. The explanation for the poor performance of the other Deep Learning models is that the size of the data set was not big enough to enable them to perform higher. In addition to this, the classic classifiers used in this paper often perform better for smaller data sets. Regarding the prediction of unanimity, our approach also reaches higher performance by using classic supervised learning algorithms for an imbalanced data set; *e.g.*, Random Forest reached an F1-score of 84.1%. When balancing the data set, all the Deep Learning models performed better than the classic classifiers with an F1-score of ∼98%.

Furthermore, our approach is easy to use as it requires that a user provide only a case description to generate predictions regarding decision outcome and unanimity. This information is relevant for attorneys, judges, and other legal professionals as it provides practical support for their work. Moreover, our contribution also includes a working prototype that can be configured for other languages and data sets.

Our contribution lays the foundation for substantial future work. For instance, our methodology can be applied to more granularly classified data sets by customizing the data by judge, law subject, and court level, among other characteristics, which will probably achieve more accurate results compared to the broad nature of the data sets used in this paper. Future investigations might also consider comparing our results with those of legal experts, as per by Ruger et al. (2004) and by current legaltech companies such as Case Crunch and LawGeex (https://www.artificiallawyer.com/2018/02/26/lawgeex-hits-94-accuracy-in-nda-review-vs-85-for-human-lawyers/).

Other future work includes investigating whether taking advantage of existent Named-entity recognition models for Brazilian law documents Luz de Araujo et al. (2018) improves the prediction quality. Furthermore, the assessment of the proposed method can be performed on other data sets, such as the European Court of Human Rights for instance. Ultimately, future work also includes evaluating our methodology by considering the data set as a time series. For this, it is necessary to rely on a larger data set that holds legal cases whose publication dates span over two years or more.

## Supplemental Information

10.7717/peerj-cs.904/supp-1 Supplemental Information 1 Reproducible Research Instructions. All steps to reproduce the results in this research.

10.7717/peerj-cs.904/supp-2 Supplemental Information 2 Legal decision data set. Ementa (summary) decisions from the Tribunal de Justiça de Alagoas, the State Supreme Court of Alagoas (Brazil), and their metadata. The file format is CSV and its separator (delimiter) is “<=>”.

10.7717/peerj-cs.904/supp-3 Supplemental Information 3 Methodology source code. We used this Python program for Steps 4, 5, 6, 7, 8, and 9 of our methodology. The Web scraper, regarding the Steps 1, 2, and 3 of our methodology, are under JusPredict (https://www.juspredict.com.br) Intellectual Property.

## Additional Information and Declarations

### Data Availability

The following information was supplied regarding data availability: All the instructions and materials (source-code and data) to reproduce the experiments are publicly available in GitHub: https://github.com/proflage/predicting-brazilian-court-decisions.
